# Delving into the Complexity of Valproate-Induced Autism Spectrum Disorder: The Use of Zebrafish Models

**DOI:** 10.3390/cells13161349

**Published:** 2024-08-14

**Authors:** Diletta Camussi, Valentina Naef, Letizia Brogi, Stefania Della Vecchia, Maria Marchese, Ferdinando Nicoletti, Filippo M. Santorelli, Rosario Licitra

**Affiliations:** 1 Department of Neurobiology and Molecular Medicine, IRCCS Stella Maris Foundation, 56128 Pisa, Italy; diletta.camussi@fsm.unipi.it (D.C.); valentina.naef@fsm.unipi.it (V.N.); stefania.dellavecchia@fsm.unipi.it (S.D.V.); maria.marchese@fsm.unipi.it (M.M.); 2 Bio@SNS, Department of Neurosciences, Scuola Normale Superiore, 56126 Pisa, Italy; letizia.brogi@sns.it; 3 Department of Physiology and Pharmacology Vittorio Erspamer, “La Sapienza” University of Rome, 00185 Rome, Italy; ferdinando.nicoletti@uniroma1.it; 4 IRCSS Neuromed, “La Sapienza” University of Rome, 86077 Pozzilli, Italy; 5 Department of Veterinary Sciences, University of Pisa, 56124 Pisa, Italy

**Keywords:** autism spectrum disorder, valproate, valproic acid, zebrafish, autism models, microglia, neuroinflammation, oxidative stress, mitochondrial respiration

## Abstract

Autism spectrum disorder (ASD) is a multifactorial neurodevelopmental condition with several identified risk factors, both genetic and non-genetic. Among these, prenatal exposure to valproic acid (VPA) has been extensively associated with the development of the disorder. The zebrafish, a cost- and time-effective model, is useful for studying ASD features. Using validated VPA-induced ASD zebrafish models, we aimed to provide new insights into VPA exposure effects during embryonic development and to identify new potential biomarkers associated with ASD-like features. Dose–response analyses were performed in vivo to study larval phenotypes and mechanisms underlying neuroinflammation, mitochondrial dysfunction, oxidative stress, microglial cell status, and motor behaviour. Wild-type and transgenic *Tg(mpeg1:EGFP)* zebrafish were water-exposed to VPA doses (5 to 500 µM) from 6 to 120 h post-fertilisation (hpf). Embryos and larvae were monitored daily to assess survival and hatching rates, and numerous analyses and tests were conducted from 24 to 120 hpf. VPA doses higher than 50 µM worsened survival and hatching rates, while doses of 25 µM or more altered morphology, microglial status, and larval behaviours. VPA 50 µM also affected mRNA expression of inflammatory cytokines and neurogenesis-related genes, mitochondrial respiration, and reactive oxygen species accumulation. The study confirmed that VPA alters brain homeostasis, synaptic interconnections, and neurogenesis-related signalling pathways, contributing to ASD aetiopathogenesis. Further studies are essential to identify novel ASD biomarkers for developing new drug targets and tailored therapeutic interventions for ASD.

## 1. Introduction

Autism spectrum disorder (ASD) is a neurodevelopmental condition characterized by deficits in social communication and repetitive patterns of behaviour [1]. Impaired motor activity and abnormal sensitivity to sensory stimuli have also been described in ASD [2]. Recent evidence suggests that autism has a global prevalence of approximately 65/10,000, with the highest rates reported in the USA and South Korean populations [3]. ASD is currently known to be a multifactorial disorder with several identified risk factors, both genetic and non-genetic [4]. In some cases, ASD symptomatology is part of a genetically determined syndrome, such as Rett syndrome, Angelman syndrome, tuberous sclerosis, and neurofibromatosis [5]. However, a large proportion of ASD cases are idiopathic, and only about 20% are caused by known genetic mutations [6]. In addition, diagnostic criteria for ASD are fairly broad due to the wide variability of types, timing, and severity of symptoms [7].

Approximately 100 genes among more than 800 identified as risk-associated have been related to the development of ASD [8,9]. Several of these genes are involved in pathways associated with gene expression regulation, chromatin remodelling, and neuro- and synaptogenesis [10,11]. Mounting evidence also links immune system dysregulation and neuroinflammation to the aetiopathogenesis of ASD, with chronic immunological brain dysfunction being identified both in humans with autism and in animal models of the disease [12,13,14]. Interestingly, 80 of these genes are expressed in the amygdala, a brain region implicated in the social component of ASD [15].

Neuroinflammation is a complex process involving glial cells (such as microglia and astrocytes) and the production of inflammatory cytokines [16,17]. In detail, microglia are the main resident immune system cells in the central nervous system (CNS) [18], and they are activated to counteract detrimental stimuli, such as infection [19,20]. Beyond their significant role in triggering and sustaining the neuroinflammatory response [21,22], microglia play a key role in neurogenesis, synaptogenesis, and synaptic regulation [23], reducing unnecessary synaptic connections early in life and, therefore, potentially becoming an important player in ASD pathogenesis [24,25].

Although a significant part of ASD susceptibility can be explained by genetics, environmental factors such as maternal and paternal age, preeclampsia, and maternal infection and/or drug use during pregnancy also play a role [26]. Among these factors, prenatal exposure to valproic acid or valproate (VPA) has been extensively linked to the development of ASD [27]. Exposure to VPA during the first trimester of pregnancy is also associated with an increased risk of congenital abnormalities, including neural tube defects and cardiac, craniofacial, and skeletal abnormalities [28,29,30,31]. Notably, multiple reports showed a higher risk of fetal abnormalities when the maternal VPA doses were above 1000 mg/day or when blood concentrations were above 70 µg/mL, which corresponds to 485 µM [32]. VPA is a short-chain fatty acid antiepileptic drug used to treat seizures and mood disorders [33]. Prenatal exposure to VPA, resulting in altering GABAergic system homeostasis, modulating voltage-gated sodium and calcium channels, and inhibiting N-methyl-D-aspartate-induced transient depolarisation and glutamatergic activity, affects several neurodevelopmental pathways [33,34,35,36]. Furthermore, VPA suppresses histone deacetylase activity, affecting gene expression and thus leading to transcription inhibition [37,38]. This interference with gene expression has been associated with changes in neural plasticity, synaptic transmission, neurogenesis, apoptotic processes, neuroinflammation, and mitochondrial metabolism [39,40,41], all mechanisms found to be impaired in ASD [42]. It has, therefore, recently been suggested that VPA may play a role in increasing oxidative stress and disrupting various physiological processes, including neurodevelopment [43].

Various animal models, such as primates, rodents, birds, and more recently, the zebrafish (*Danio rerio*), have been used to study ASD and to elucidate the biological mechanisms of action of VPA [44,45,46,47]. These models have exhibited features resembling the human ASD phenotype, including motor and social behavioural deficits, increased anxiety, and developmental abnormalities [48,49,50]. The zebrafish, in particular, is emerging as a cost- and time-effective organism for reproducing and studying the features of autism [51,52,53] and for identifying the factors leading to the development of ASD [2]. Because of the developmental, anatomical, functional, and genetic similarities between the zebrafish and mammalian nervous systems, the zebrafish is indeed a promising model organism in neuropharmacological research [54]. Since the development of the zebrafish embryo is similar to the early stages of human embryonic development, the zebrafish in vivo model can be used as a chemical screening tool to investigate the interplay between genes and the environment. Other advantages of this highly social and genetically tractable organism that make it useful for translational studies include its external development, which makes it easy to reproduce VPA exposure during pregnancy by adding VPA to the medium in which the zebrafish embryos and larvae grow [50]; the transparency of the zebrafish embryo, which facilitates the use of non-invasive imaging techniques; and the availability of genetic and non-genetic lines for studying ASD [52,55,56,57,58].

Zebrafish embryonically exposed to VPA (to model ASD) display anxiety, deficits in social interaction, and increased neuroinflammatory profiles; they also exhibit autistic locomotor phenotypes and several somatic and nervous system developmental abnormalities [59,60,61,62]. Furthermore, VPA administration in zebrafish has been shown to impair serotonergic and dopaminergic system development [63,64].

In this paper, we used validated VPA-induced ASD zebrafish models [2] to study the larval phenotype, investigate the presence of neurodevelopmental impairments, and highlight possible mechanisms underlying these features by exploring neuroinflammation and neurogenesis-related gene expression, as well as markers of oxidative stress and mitochondrial dysfunction. We focused on understanding the possible role of microglial activation in ASD by analysing microglial cells in vivo in a transgenic zebrafish line expressing enhanced green fluorescent protein (EGFP) in microglial and macrophagic cells. Finally, we explored the potential impacts of VPA treatment on cardiac and optokinetic responses to evaluate possible repercussions on the cardiovascular and visual systems. The novelty of the research relies on providing new insights into the aetiology of ASD induced by VPA exposure during embryonic development and identifying new potential biomarkers that could be associated with ASD-like features through a comprehensive battery of molecular and behavioural tests.

## 2. Materials and Methods

### 2.1. Ethical Regulations

The experimental procedures were conducted under the supervision and with the approval of the Animal Care and Use Committee of the IRCCS Stella Maris Foundation (Pisa, Italy) and in compliance with European Directive No. 63 of 22/09/2010 on the protection of animals used for scientific purposes. Throughout the study, zebrafish embryos and larvae up to five days post-fertilization were used, in line with the “3Rs” guiding principles for research involving the use of laboratory animals [65].

### 2.2. Chemicals

Valproic acid sodium salt (CAS-No: 1069-66-5), tricaine (CAS-No: 886-86-2), (+)-tubocurarine chloride pentahydrate (CAS-No: 6989-98-6), 1-phenyl-2-thiourea (PTU, CAS-No: 103-85-5), low-gelling-temperature agarose (CAS-No: 39346-81-1), and methyl cellulose (CAS-No: 9004-67-5) were obtained from Sigma-Aldrich (St. Louis, MO, USA). A 100 mM stock solution of VPA was prepared by dissolving VPA in ultra-pure water. Experimental solutions were freshly prepared in standard fish medium, which consisted of osmotic water with 60 mg per litre of “Instant Ocean^®^” sea salt (Spectrum Brands, Blacksburg, VA, USA). Drug doses were chosen on the basis of preliminary results and data obtained by Chen et al. [2], ranging from 5 to 500 µM.

### 2.3. Animals

Adult wild-type AB strain (WT-AB) and transgenic *Tg(mpeg1:EGFP)* zebrafish from the Department of Neurobiology and Molecular Medicine of the IRCCS Stella Maris Foundation were used. Parental fish were maintained according to standard procedures [64], and once spawned, eggs were collected and incubated at 28.5 °C in Petri dishes (Ø 10 cm, 40 eggs per Petri) filled with 50 mL of egg water until 6 h post-fertilisation (hpf). Wild-type AB fish were used for preliminary toxicity analysis to assess the effects of VPA on locomotor behaviour, morphology, mitochondrial respiration rate, and reactive oxygen species (ROS) accumulation, as well as for gene expression analysis. Transgenic fish were used primarily to study microglial status in the CNS in vivo. Microglia were evaluated by pre-treating transgenic embryos at 24 hpf with 0.003% PTU to inhibit pigmentation, as described elsewhere [66]. In this PTU-treated line, we also performed two tests in which larval transparency is crucial: optokinetic response (OKR) and cardiac function tests.

### 2.4. Experimental Design

The experimental design had two phases. Initially, various doses of VPA (ranging from 5 to 500 µM) were administered to WT-AB embryos by means of water exposure in order to confirm the minimal effective concentration and to evaluate dose–response toxicological effects on zebrafish development. Subsequently, *Tg(mpeg1:EGFP)* zebrafish embryos were exposed to non-lethal doses of VPA (ranging from 5 to 50 µM) to assess VPA effects on microglia, OKR, and heart rate. Treatments started at 6 hpf (gastrula period, at approximately 50% epiboly stage) and lasted until 120 hpf. All experiments were replicated thrice (40 individuals per replicate, 120 per treatment) to ensure the reliability and consistency of results.

### 2.5. Measurements and Analyses

Throughout the experimental period, several parameters were measured and analysed. Embryos and larvae were monitored daily to assess survival and hatching rates. The following is a comprehensive overview of the tests and analyses conducted in the course of the experimental trial.

#### 2.5.1. Test of Embryo Locomotor Behaviour

Given the considerable sensitivity of the developing nervous system to drug exposure, we first implemented a standardised behavioural test, namely, the tail-coiling test, as described by Licitra et al. [50], to evaluate the effects of the compound on neurotransmission. Briefly, movements of unhatched embryos at 24 hpf were directly video-recorded in their rearing Petri dishes using a Leica M205FA stereomicroscope (Leica, Wetzlar, Germany) connected to DanioScope software version 1 (Noldus Information Technology, Wageningen, The Netherlands) for video analysis.

#### 2.5.2. Morphological Evaluation

At 120 hpf, all groups from both lines underwent morphological evaluation, which included measurements of body length, eye area, swim bladder area, and the presence/absence of pericardial oedema. Larvae were first anaesthetised by immersion in a water bath containing tricaine (0.168 mg/mL) and then individually placed in a transparent cavity slide under the Leica M205FA stereomicroscope for image capture. Data analysis was performed using DanioScope software. On the basis of morphological characteristics (as well as survival and hatching rates), we identified two phenotypes of interest: mild (animals administered VPA 25 µM) and moderate (VPA 50 µM).

#### 2.5.3. Tests of Larval Locomotor Behaviour

Additional behavioural analyses were conducted in these mild and moderate VPA phenotypes to further explore the biological effects of VPA on sensorimotor system development. The locomotor behaviour analysis included an assessment of swimming under different light conditions (visual motor response test) and following a stressful stimulus (startle response test). The visual motor response test was performed on WT-AB hatched larvae at 120 hpf using 96-well plates (1 larva per well in 300 µL of medium) placed in the DanioVision apparatus connected with EthoVision XT17 software (Noldus Information Technology, Wageningen, The Netherlands). Larval locomotion was analysed for 20 min in the dark, followed by 10 min in the light and another 20 min in the dark. By exploring larval behavioural phenotypes under both light and dark conditions, we were also able to evaluate sensorimotor function, which is dependent on neuro-optic development, as suggested by Tuz-Sasik et al. [67]. The startle response assay was used to assess fish stress reaction. By using the DanioVision system connected with EthoVision XT17 software, the locomotor behaviour of WT-AB zebrafish larvae could be evaluated before and after a stressful stimulus automatically generated by a tapping device (stimulus intensity was set at the maximum level: 8/8). Locomotion was measured for a total of 20 min: 10 min in normal conditions (undisturbed larvae) and 10 min after the stressful stimulus.

#### 2.5.4. Mitochondrial Respiration Assay and ROS Analysis

In the two phenotypes of interest, mitochondrial respiration and ROS analysis were conducted following the protocol outlined by Naef et al. [68]. The first assay was performed at 120 hpf using the XF24 extracellular flux analyser (Seahorse Bioscience, North Billerica, MA, USA). ROS levels were assessed using an in vivo carboxy-H2DCFDA fluorescent probe (Abcam, Cambridge, MA, USA) at 30 hpf, according to Schindelin et al. [69]. To this end, a lateral image of each larva was captured using a fluorescence microscope, and the fluorescence intensity in the selected region of interest was quantified using ImageJ 64 software (Fiji, Los Angeles, CA, USA).

#### 2.5.5. RNA Isolation and Quantitative Reverse Transcription Polymerase Chain Reaction (qRT-PCR)

Total RNA was extracted from 30 larvae per group at 120 hpf using Quick RNA miniprep (ZymoResearch, Irvine, CA, USA) according to the manufacturer’s instructions. cDNA synthesis and qRT-PCR analysis were performed as described by Licitra et al. [70]. To quantify relative mRNA expression, we used the Mic Real-Time PCR System (Bio Molecular Systems, Upper Coomera, Australia) and the comparative ΔCt method. All data were normalised to the expression of the housekeeping gene, *β-actin*. Supplementary Table S1 lists the sequences of the primers used.

#### 2.5.6. Microglia Analysis

To visualise active microglia in the nervous system, living larvae were anaesthetised using tubocurarine (2 mM) and then embedded in 200 µL of 1.2% low-melting-point agarose. To slow down dehydration, 200 µL of 5% methyl cellulose was used to cover the agarose drop containing the larva. Image acquisition was performed using an LSM 700 confocal microscope (Zeiss, Jena, Germany). Z-stack images of *mpeg1:EGFP* larvae were acquired with a step size of 2 μm using a 20× objective and 220 μm thick sections. Two independent operators manually counted the number of microglial cells in the larval whole brains. Microglial cell sphericity was quantified using ImageJ 64 software with the MorphoLibJ plugin [71]. To analyse the sphericity of microglial cells, six different areas were selected per individual: one in the telencephalon, two in the optic tectum, and two in the hindbrain (Figure S1).

#### 2.5.7. Heart Rate

At 72 hpf, heart rate measurements were performed in all transgenic embryo groups, following the procedure described by Runfola et al. [72]. Briefly, unsedated hatched larvae were individually placed in a transparent cavity slide under the stereomicroscope, and 15 s videos of the pericardial area of each fish were recorded at 30 frames per second. Heart rate data analysis was conducted using DanioScope software.

#### 2.5.8. Optokinetic Response Test

The OKRs of zebrafish larvae were measured according to Brockerhoff [73] to evaluate fish visual and oculomotor function and to characterise related neural pathway defects [74,75]. This assay allows the measurement of larval eye movements (rapid resetting saccades) under visual stimulation. To this end, larvae were partially immobilised in a 5% methyl cellulose solution on the surface of a reversed Petri dish (Ø 3.5 cm) placed in the centre of the drum of the VisioBox (ViewPoint Behavior Technology, Lyon, France), in which alternating black and white vertical stripes were digitally projected. Larvae were placed in a dorsal-up position and in an X shape to prevent them from touching or seeing each other (Figure S2). The number of eye saccades was measured using the specific Viewpoint automated software (PHI Visualize, version 5.29.0.180) (Physical Electronics, Chanhassen, MN, USA).

### 2.6. Statistical Analysis

Survival and hatching rates were evaluated using the Mantel–Cox log-rank test. Other data were analysed using either parametric or non-parametric methods depending on the distribution of the response variable, assessed by means of the Shapiro-Wilk test. The Kruskal–Wallis test with Dunn’s multiple comparisons test was used to analyse embryo burst activity, morphology, larval heart rate, and OKR. The ordinary two-way ANOVA with Tukey’s multiple comparisons test was used for the analysis of larval locomotor behaviour. The unpaired *t*-test with Welch correction was used for mitochondrial respiration, ROS, mRNA gene expression, and microglia analyses. Statistical significance was defined as *p* ≤ 0.05, and all analyses were conducted using GraphPad Prism 9 (Graph-Pad Software, San Diego, CA, USA).

## 3. Results

### 3.1. Survival and Hatching Rates: Worsened by VPA Doses Higher Than 50 µM

**Figure 1:**
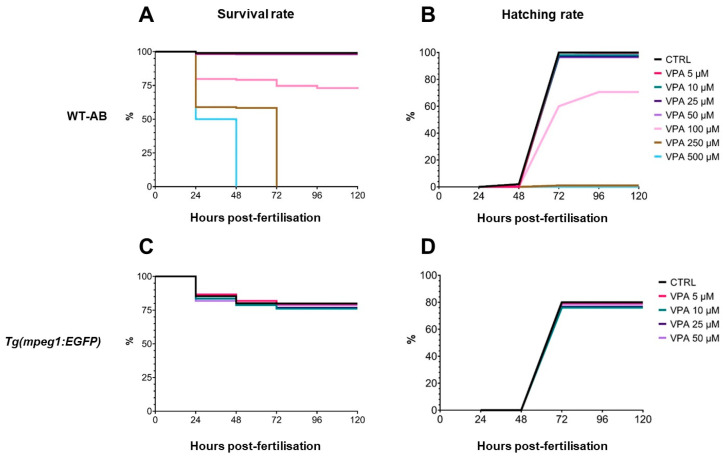
Effect of valproic acid (VPA) on zebrafish larval survival and hatching rates at up to 120 h post-fertilisation (hpf) (*n* = 120 per group). (**A**) Kaplan–Meier survival comparison in WT-AB fish showed a significant effect [log-rank (Mantel–Cox) test] of VPA treatment at 100 (*p* ≤ 0.05), 250, and 500 μM (*p* ≤ 0.0001) as compared with untreated controls (CTRL) as early as 24 hpf. (**B**) The hatching rate of WT-AB fish was significantly affected [log-rank (Mantel–Cox) test] by VPA treatment with doses of 100 (*p* ≤ 0.05), 250, and 500 μM (*p* ≤ 0.0001) at 72 hpf. (**C**,**D**) Survival and hatching rates of *Tg(mpeg1:EGFP)* transgenic fish were not influenced by any tested treatment (*p* > 0.05).

In WT-AB larvae, survival and hatching rates were similar between controls and fish treated with VPA doses of up to 50 µM (Figure 1A,B). However, when doses of 100 µM or more were used, significant worsening effects were observed. In particular, VPA 100 µM reduced survival and hatching rates by 25 and 30%, respectively. VPA doses of 250 µM or more prevented hatching and caused the death of all embryos within 72 hpf. Similarly, VPA doses of up to 50 µM did not affect survival and hatching rates in *Tg(mpeg1:EGFP)* (Figure 1C,D). Although survival and hatching rates of transgenic zebrafish were lower than those of WT ones (–20%), VPA treatment at low doses showed similar effects in both genetic lines.

### 3.2. Embryo Tail-Coiling Behaviour: Reduced by VPA Doses Higher Than 50 µM

**Figure 2:**
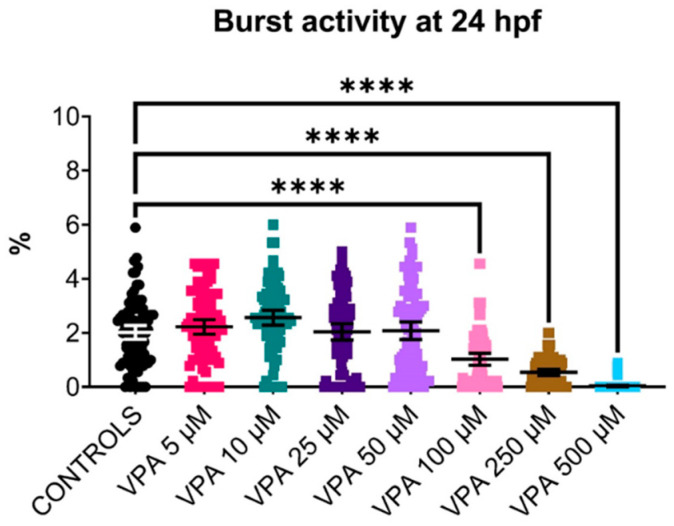
Effect of valproic acid (VPA) on WT-AB embryo burst activity at 24 h post-fertilisation (hpf) (*n* = 90 per group). The tail-coiling test results showed a significant reduction (**** *p* ≤ 0.0001, Kruskal–Wallis test) in burst activity of embryos treated with VPA at doses higher than 50 μM compared with untreated controls. Data are represented as individual values (lines indicate means ± SEM).

The tail-coiling test was performed in the WT-AB strain, and the results (Figure 2) showed similar burst activity between controls and VPA-treated embryos up to the 50 μM dose, with no significant differences observed between these groups. However, higher VPA doses significantly reduced embryo burst activity compared with controls (*p* ≤ 0.0001), suggesting a neurotoxic effect of the compound. In particular, the highest dose (500 μM) almost suppressed all spontaneous side-to-side contractions of the embryo trunk inside the chorion.

### 3.3. Morphology: Affected by VPA Doses of 25 µM or Higher

**Figure 3:**
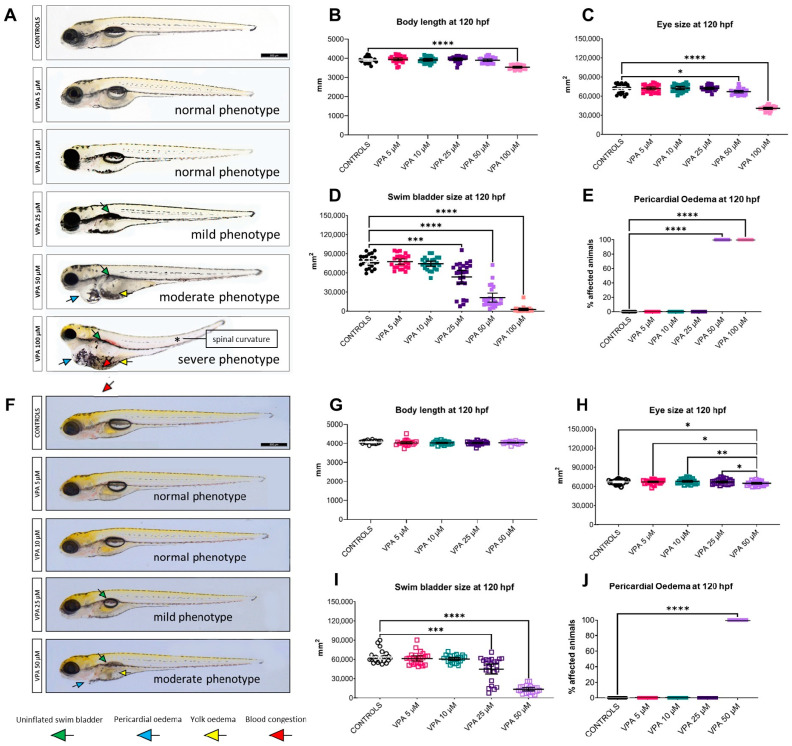
Effects of valproic acid (VPA) on larval morphology at 120 h post-fertilisation (hpf) (*n* = 24 per group). (**A**) Representative images of WT-AB untreated controls and VPA-treated larvae. (**B**–**E**) Morphological evaluation in WT-AB fish showed significant effects (* *p* ≤ 0.05, *** *p* ≤ 0.001, **** *p* ≤ 0.0001, Kruskal–Wallis test) of VPA treatment at doses higher than 25 μM as compared with untreated controls for all measured parameters. (**F**) Representative images of *Tg(mpeg1:EGFP)* untreated controls and VPA-treated larvae. (**G**–**J**). Morphological evaluation of *Tg(mpeg1:EGFP)* fish showed a significant effect (* *p* ≤ 0.05, ** *p* ≤ 0.01, *** *p* ≤ 0.001, **** *p* ≤ 0.0001, Kruskal–Wallis test) of VPA treatment at 50 μM as compared with untreated controls for eye size, swim bladder size, and presence of pericardial oedema. Data are represented as individual values (lines indicate means ± SEM).

Figure 3 shows representative images of untreated controls and VPA-treated larvae of both genetic strains at 120 hpf, together with the analysed morphological parameters. Larvae treated with VPA doses of up to 10 μM showed normal morphology (Figure 3A,F). However, the use of 25 μM VPA was associated with a significant reduction in swim bladder inflation, while partial and total failure to inflate the swim bladder was observed with doses of 50 and 100 μM, respectively (higher dose analysed only in the WT-AB specimens). In addition, both the 50 and the 100 μM VPA dose caused a decrease in larval eye size and an increase in the rate of pericardial oedema, with the highest dose also causing a significant reduction in larval body length.

### 3.4. Larval Locomotor Behaviour: Reduced by 25 µM VPA Dose and Suppressed by 50 µM VPA Dose

**Figure 4:**
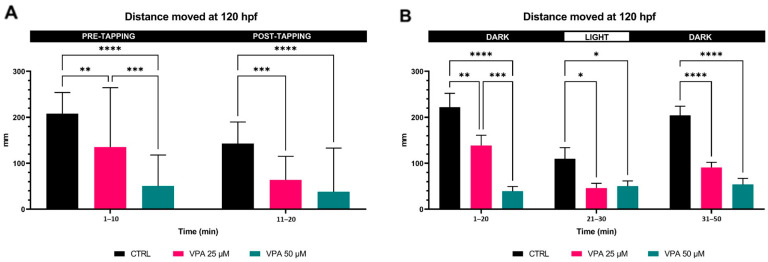
Effects of valproic acid (VPA) on locomotor behaviour in WT-AB larvae at 120 h post-fertilisation (hpf) (*n* = 24 per group). (**A**) Startle response data analysis showed significant dose-dependent reductions of larval locomotor activity, both before and after the mechanical stimulation (** *p* ≤ 0.01, *** *p* ≤ 0.001, **** *p* ≤ 0.0001, two-way ANOVA test). (**B**) Visual motor response data analysis showed significant dose-dependent reductions of larval locomotor performance in both dark and light conditions (* *p* ≤ 0.05, ** *p* ≤ 0.01, *** *p* ≤ 0.001, **** *p* ≤ 0.0001, two-way ANOVA test). Data are represented as means ± SEM.

Two behavioural tests were used to investigate the locomotor activity of larvae (Figure 4), and both involved exposing the fish to a stressful stimulus. The results of the first test (startle response test) showed that the controls and 25 μM VPA-treated fish responded similarly in terms of decreased motor activity following the tapping stimulus, whereas the larvae treated with the higher dose (50 μM VPA) did not show a significant decrease in distance travelled following the mechanical stimulus. Overall, treatment with VPA significantly reduced the locomotor performance of the larvae in a dose-dependent manner, both in the pre- and post-tapping phases (Figure 4A). The results of the second behavioural test (visual motor response) showed that both VPA doses reduced larval locomotor activity under both light and dark conditions (Figure 4B). In this case, too, controls and 25 μM VPA-treated fish responded similarly in terms of significantly decreased motor activity following the stressful stimulus (light condition), while larvae treated with the higher dose (50 μM VPA) showed suppressed locomotion in both conditions (dark and light) with any significant locomotor variation following the stressful stimulus.

### 3.5. Mitochondrial Respiration and ROS Accumulation: Altered by 50 µM VPA Dose

**Figure 5:**
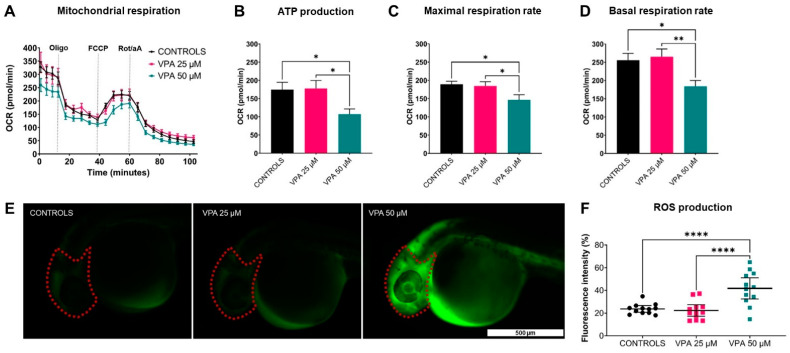
Effects of valproic acid (VPA) on WT-AB mitochondrial respiration at 120 h post-fertilisation (hpf) and on reactive oxygen species (ROS) accumulation at 30 hpf (*n* = 12 per group). (**A**) Mitochondrial respiratory analysis showed a decreased oxygen consumption rate (OCR) in 50 μM VPA-treated larvae compared with controls and 25 μM VPA-treated larvae. (**B**–**D**) ATP production and both maximal and basal respiration rates were reduced in 50 μM VPA-treated larvae compared with controls and 25 μM VPA-treated larvae (* *p* ≤ 0.05, ** *p* ≤ 0.01, *t*-test with Welch correction). Data are represented as mean ± SEM. (**E**) Representative fluorescence images of ROS production in controls and VPA-treated larvae, with red dotted lines indicating the region of interest. (**F**) Quantitative analysis of ROS production showed a significant increase in 50 μM VPA-treated larvae compared with controls and 25 μM VPA-treated larvae (**** *p* ≤ 0.0001, *t*-test with Welch correction). Data are represented as individual values (lines indicate means ± SEM).

In vivo mitochondrial respiration data revealed impaired mitochondrial bioenergetics in 50 μM VPA-treated larvae compared with controls and 25 μM VPA-treated larvae, as shown by significant reductions in ATP production and in both basal and maximal respiration rates (Figure 5A–D). Furthermore, an approximately 20% increase in ROS production was observed in 50 μM VPA-treated larvae compared with controls (Figure 5F). Taken together, these findings suggest an increment of oxidative stress in larvae treated with VPA at the higher dose.

### 3.6. mRNA Gene Expression: Altered by 50 µM Dose

**Figure 6:**
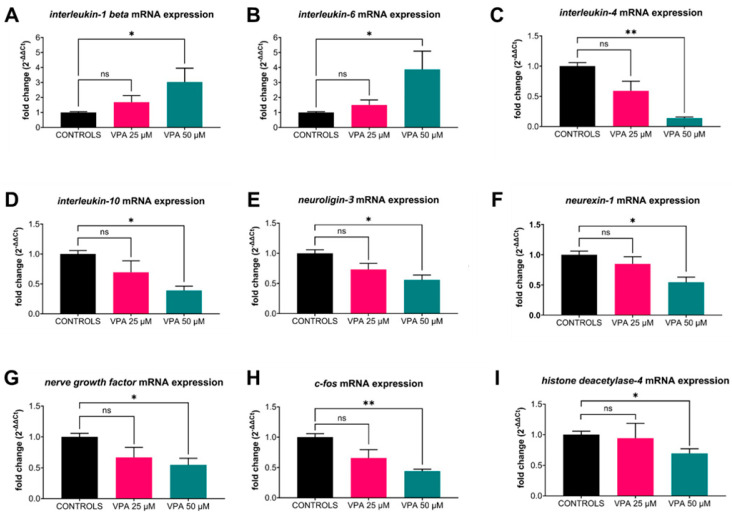
Effects of valproic acid (VPA) on WT-AB larvae mRNA gene expression values at 120 h post-fertilisation (hpf) (*n* = 90 per group). (**A**,**B**) Data analysis showed a significant upregulation of pro-inflammatory cytokines (*IL-1β* and *IL-6*) in 50 μM VPA-treated larvae compared with controls (* *p* ≤ 0.05, *t*-test with Welch correction). (**C**,**D**) Anti-inflammatory cytokines (*IL-4* and *IL-10*) were downregulated in 50 μM VPA-treated larvae compared with controls (* *p* ≤ 0.05, *t*-test with Welch correction). (**E**–**H**) Neurogenesis- and neuronal activation-related genes (*nlgn3*, *nrxn1*, *ngf*, and *c-fos*) were downregulated in 50 μM VPA-treated larvae compared with controls (* *p* ≤ 0.05 or ** *p* ≤ 0.01, *t*-test with Welch correction). (**I**) Expression of the VPA-target gene *(hdac4)* was downregulated in 50 μM VPA-treated larvae compared with controls (* *p* ≤ 0.05, *t*-test with Welch correction). Data are represented as means ± SEM. Abbreviations: ns = not significant.

Gene expression analysis (Figure 6) showed increases in the pro-inflammatory cytokines *interleukin-1 beta (IL-1β)* and *interleukin-6 (IL-6)* and decreases in anti-inflammatory markers *interleukin-4 (IL-4)* and *interleukin-10 (IL-10)* in response to the administration of 50 μM VPA. Neurogenesis- and neuronal activation-related genes *neuroligin-3 (nlgn3)*, *neurexin-1 (nrxn1)*, *c-fos*, and *nerve growth factor (ngf)* were also downregulated in 50 μM VPA-treated larvae compared with control and 25 μm VPA-treated larvae. *Histone deacetylase-4 (hdac4)*, which is the VPA target gene, was downregulated in 50 μM VPA-treated larvae compared with the control and 25 μM VPA-treated groups.

### 3.7. Microglial Cell Status: Altered by 25 and 50 µM Doses of VPA 

**Figure 7:**
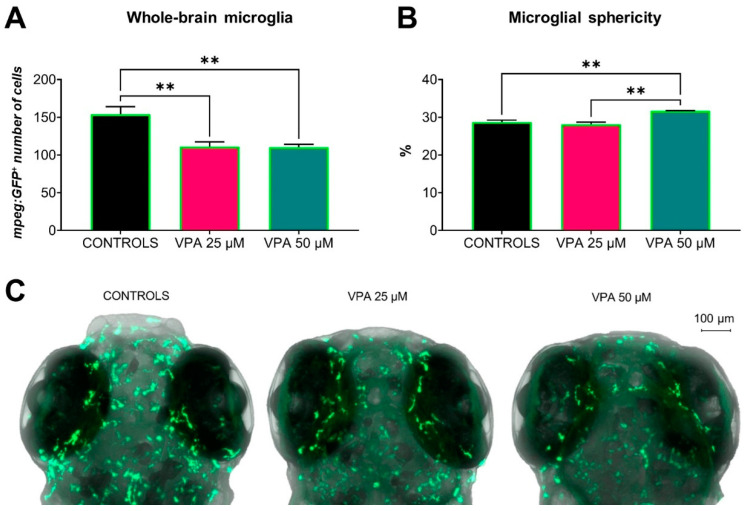
Effects of valproic acid (VPA) on *Tg(mpeg1:EGFP)* microglial cell status at 120 h post-fertilisation (*n* = 3 per group). (**A**) Whole-brain analysis showed a significantly decreased number of microglia in VPA-treated larvae compared with controls (** *p* ≤ 0.01, *t*-test with Welch correction). (**B**) Analysis of microglial cell morphology showed significantly increased sphericity in 50 μM VPA-treated larvae compared with controls and 25 μM VPA-treated larvae (** *p* ≤ 0.01, *t*-test with Welch correction). Data are represented as means ± SEM. (**C**) Representative confocal images of microglial cell morphology in controls and VPA-treated larvae.

The in vivo whole-brain analysis of the transgenic line showed significant decreases in the number of microglia in the two VPA-treated groups compared with the controls (Figure 7A). Microglia can be classified into two types: classical (M1) and alternative (M2), although there is a continuum of intermediate phenotypes. Analysis of microglial morphology showed a significant increase in the rate of microglial cell sphericity in 50 μM VPA-treated larvae (increase in the M1-like phenotype) compared with controls and 25 μM VPA-treated larvae (Figure 7B), suggesting an increase in pro-inflammatory microglial activity in the group treated with the higher VPA dose.

### 3.8. Heart Rate: All VPA Doses Caused Increased Heart Rate

**Figure 8:**
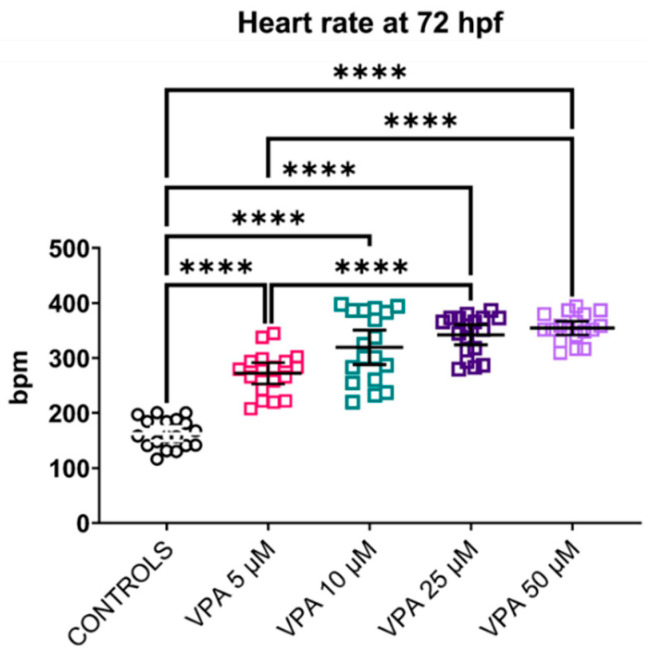
Effects of valproic acid (VPA) on heart rate in *Tg(mpeg1:EGFP)* larvae at 72 h post-fertilisation (hpf) (*n* = 24 per group). Heart rate analysis showed increased beats per minute (bpm) in all larvae treated with VPA (**** *p* ≤ 0.0001, Welch’s ANOVA test) compared with untreated controls. Data are represented as individual values (lines indicate means ± SEM).

The heart rate analysis suggested that all VPA treatments caused tachycardia in transgenic zebrafish larvae at 72 hpf (Figure 8). Heartbeats per minute were significantly increased in all VPA-treated fish compared with the untreated controls (*p* ≤ 0.0001). However, it is worth noting that the VPA 25 and 50 μM doses caused a significant increase in heart rate compared with what was observed in the group treated with VPA 5 μM.

### 3.9. Optokinetic Response: Altered by 25 and 50 µM Doses of VPA 

**Figure 9:**
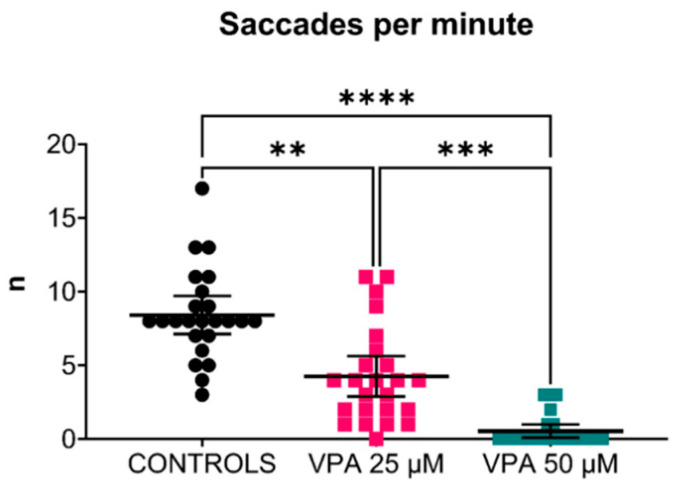
Effects of valproic acid (VPA) on optokinetic response in *Tg(mpeg1:EGFP)* larvae at 120 h post-fertilisation (*n* = 24 per group). Data analysis showed a significantly decreased number of saccades per minute in VPA-treated larvae compared with controls (** *p* ≤ 0.01, *** *p* ≤ 0.001, Kruskal–Wallis test). The difference was greater between the controls and the 50 μM VPA-treated larvae (**** *p* ≤ 0.0001, Kruskal–Wallis test). Data are represented as individual values (lines indicate means with 95% confidence interval).

The number of eye saccades under visual stimulation was significantly reduced in VPA-treated transgenic zebrafish larvae compared with controls at 120 hpf. Larvae treated with the 25 μM VPA dose showed approximately half the number of eye saccades per minute recorded in controls, while saccades in 50 μM VPA-treated larvae were almost abolished (Figure 9).

## 4. Discussion

Animal models are invaluable tools in unravelling the complex mechanisms underlying ASD and for exploring potential biomarkers and therapeutic strategies. The zebrafish, due to its genetic, neurobiological, and behavioural similarities to humans, offers a powerful platform for elucidating the gene–environment interplay involved in the pathogenesis of ASD. Previous studies have shown that by exposing zebrafish embryos to VPA (from 5 to 2000 µM) at critical stages of development, it is possible to simulate human prenatal exposure to VPA and reproduce key features of ASD, such as social and cognitive deficits and neurobiological alterations [2,63,76,77]. This study, focusing on two ASD-like zebrafish phenotypes (one mild and the other moderate, obtained by exposing zebrafish embryos to 25 μM VPA and 50 μM VPA, respectively), confirmed previous observations and provided comprehensive insights into the neurodevelopmental and behavioural effects of VPA exposure in zebrafish larvae.

In line with several studies, we observed that exposure to VPA during embryonic development can significantly impact fish survival. Chronic exposure to high doses of VPA (25–100 μM) leads to reduced embryo hatching and increased mortality [2,47,78,79]. In addition to VPA concentration, the duration of exposure and the stage of embryo development at the time it occurs also affect toxicological results. Data documenting the effects of VPA in zebrafish are, therefore, characterised by a high degree of variability. The route of administration of the molecule undoubtedly plays a central role, too. VPA can be dissolved in water or in other solvents, such as ethanol or DMSO, both already known to be toxic to fish [50]. In line with our results on survival and hatching rates, previous authors did not observe significant effects when using VPA dissolved in water at doses of up to 50 μM [60,78], although sub-lethal effects were detected at a dose of 82 μM [78]. Conversely, some authors reported no effect on mortality even at 1500 μM [2], while others observed a significant effect on survival and hatching starting from VPA doses of 20–30 μM [47], with survival totally suppressed at 640 μM [80].

In our study, biometric evaluation demonstrated that exposure to VPA, particularly at doses of 25 µM or more, significantly affects the morphology and development of zebrafish larvae, reproducing the human malformations linked to VPA exposure during pregnancy. Interestingly, however, larvae treated with up to 10 µM of VPA displayed normal morphology, confirming a dose-dependent effect of the compound on morphological features. Exposure to 25 µM resulted in a significant reduction in swim bladder inflation, related to reduced development. Moreover, higher doses of VPA (50 and 100 µM) led to partial and total failure to inflate the swim bladder, respectively, indicating more severe disruption of embryonic development. These findings are consistent with previous evidence that exposure to non-lethal concentrations of VPA during early embryonic stages can induce morphological alterations, such as uninflated swim bladder, pericardial oedema, and widespread developmental abnormalities, particularly at doses above 500 µM [2,80,81]. Overall, these findings underscore the teratogenic effects of VPA on zebrafish embryo morphology and highlight the importance of considering dose-dependent responses when assessing the developmental toxicity of VPA. On the basis of these toxicological and morphological observations, we focused on the mild and moderate VPA-induced phenotypes (the groups treated with 25 and 50 µM, respectively). The groups treated with doses lower than 25 µM did not show any significant differences versus the untreated control group, while higher doses caused severe toxic effects. The investigation of cardiac parameters showed that VPA caused tachycardia at both the tested doses, as described in our previous research [50]. Cardiac anomalies are described in human newborns exposed to VPA during prenatal development [82]. However, the available literature data on the effect of VPA on zebrafish cardiac parameters are extremely limited. In a study where VPA doses greater than 100 µM were used (sub-lethal concentration), a robust decrease in heartbeats per minute was observed [83]. In the current work, treatment with 100 µM VPA caused severe pericardial oedema and trunk blood congestion, compromising the whole cardiac system. Some authors suggest that histone deacetylase inhibition, through aberrant gene expression during cardiogenesis, might be the underlying cause of cardiac system dysfunction [83]. Further in-depth investigation is needed to clarify how VPA and ASD are linked to cardiac impairments.

Although there appear to be no available data on the OKR in zebrafish larvae treated with VPA or in other fish models of ASD, some authors have demonstrated that sub-lethal levels of VPA decrease the retinotectal projection area in the optic tectum [77], potentially compromising the visual system of exposed fish. Our OKR findings suggest that VPA can reduce or even abolish visual function in these fish, which showed no significant oculomotor responses to visual stimulation after treatment with high doses of VPA.

We confirmed that VPA impacts the behavioural phenotype of zebrafish embryos and larvae, finding tail coiling to be reduced by exposure to the 100 µM dose of VPA and totally suppressed by 500 µM. Similar results were reported by Joseph et al. [47], who described a reduction in coiling behaviour after exposure to 80 μM VPA, with no coiling produced by embryos exposed to doses of 320 μM or more. Changes in burst activity are probably linked to perturbations in neurogenesis-related signalling pathways together with abnormal neuronal development in the hindbrain, leading to impaired neuronal hyperexcitability [79]. The locomotor behaviour of swimming larvae was also found to be significantly affected by exposure to VPA, both in normal conditions and following stressful stimuli (dark–light transition and tapping), with distinct responses observed at different doses. In all vertebrates, locomotor behaviour represents a highly conserved function, primarily generated by a network of neurons (Central Pattern Generators) located in the spinal cord [76]. These neurons are mainly constituted by excitatory glutamatergic motor neurons modulated by inhibitory GABAergic/glycinergic interneurons. Specifically, the fine-tuned action of such neurons provides zebrafish with a coordinated muscle contraction, which enables their swimming. Also, proper locomotor behaviour is reached by sensory system afferences, which display a modulatory role. Moreover, according to circumstantial needs, these systems are modulated by different classes of neurotransmitters, mainly represented by the endocannabinoid system (modulation of adult swimming and escape circuits), the dopaminergic system (modulation of overall swim levels according to developmental stages), and the serotoninergic system (increasing swimming frequency in the larval stage and decreasing swimming frequency during the juvenile/adult stage) [84]. In addition, in normal laboratory conditions, larval locomotor activity is influenced by light conditions and transitions between light and dark environments [50], as well as other stressful stimuli, both acoustic and mechanical. In line with our results, several studies have reported impaired dark-flash responses in VPA-exposed larvae, suggesting deficiencies in the underlying neural circuitry responsible for regulating locomotor behaviour [63,77,85,86]. Motor impairments have been identified as early signs of ASD [87]. In zebrafish larvae, VPA exposure influences both excitatory and inhibitory neurotransmitter systems, contributing to the observed alterations in locomotor response patterns [77]. This dysregulated neural signalling may affect various behavioural aspects and motor functions. For instance, the disruption of dopaminergic and GABAergic pathways, known to be modulated by VPA [63,64], may contribute to the altered locomotor responses observed in affected larvae.

In the second part of the study, we explored the possible mechanisms through which VPA might produce the altered phenotype and neurodevelopmental impairment that we observed in zebrafish larvae.

First, we found that VPA treatment led to significant alterations in micro-oxygraphy parameters, with a reduction in energy production and an increase in oxidative stress. This result is in line with recent evidence suggesting that VPA induces mitochondrial dysfunction and, consequently, the generation of ROS [88]. Interestingly, prenatal VPA exposure in rats seems to induce ASD-like behaviours, also via ROS accumulation and the activation of pro-apoptotic pathways [89]. This cellular impairment may affect physiological neuronal maturation and alter the developing brain [90]. Furthermore, impairments in mitochondrial function have been associated with several neuropsychiatric disorders, including autism; indeed, a recent meta-analysis emphasised the strong link between ASD and mitochondrial metabolism [91] that has been highlighted by the detection of numerous mitochondrial dysfunction biomarkers in the peripheral blood of people with autism [7,92]. In summary, given the high mitochondrial presence in muscle and neuron cells, which are primarily responsible for locomotor activity, any impairment of bioenergetic dynamics could contribute to the observed reduced swimming performance. However, the exact mechanisms by which mitochondria contribute to the pathophysiology of neurodevelopmental disorders remain poorly understood.

Second, we explored the effect of VPA on neuronal gene expression and microglial activation since ASD animal model [93,94] and post-mortem human brain [95] studies have revealed the downregulation of genes involved in synaptic function and neuronal survival, as well as the upregulation of immune-related and pro-inflammatory genes. We confirmed that VPA exposure downregulates genes related to neurogenesis and neuronal survival (*nlgn3*, *nrxn1*, *c-fos*, and *ngf*), findings that support the hypothesis that VPA could act directly on the expression of genes classically related to ASD, like *nlgn3* and *nrxn1* [96]. In accordance with previous studies [97,98], the expression of the VPA target gene, *hdac4*, was found to be downregulated. The inhibition of *hdac4* has been shown to be essential in inducing the behavioural and brain developmental alterations observed in VPA-related ASD [99]. 

Regarding our analysis of neuroinflammation, we observed the increased expression of pro-inflammatory cytokines (*IL-1β* and *IL-6*) and the reduced expression of anti-inflammatory cytokines (*IL-4* and *IL-10*) in zebrafish larvae exposed to VPA versus controls. Morphological analysis of microglial cells showed a reduction in the number of ramified microglial cells and an increase in the number of spherical ones in larvae exposed to VPA versus control larvae. The increase in pro-inflammatory cytokines, reduction in anti-inflammatory cytokines, reduction in ramified microglia, and increased sphericity of the microglial cells seemed to indicate a switch towards the M1 or pro-inflammatory phenotype of microglia in larvae exposed to VPA compared with controls. In simple terms, microglia, depending on their activation state, can assume two different phenotypes, M1-like or M2-like, the former being associated with pro-inflammatory cytokine production, ROS production, and neuronal damage, and the latter creating a favourable environment for the physiological functions of neurons, supporting the release of anti-inflammatory cytokines and neurotrophic factors [20,100]. Consistent with our findings, Solso et al. [101] hypothesised that an increase in the M1-like phenotype and a concomitant decrease in branching microglia may underlie altered synaptic homeostasis, causing dysfunction in synaptic interconnections and redundant cortical networks in the brains of people affected by ASD. In recent work [88], exposure to an organophosphate showed similar results in zebrafish larvae, with an increase in the sphericity of microglia and a decrease in the average number of ramified microglia. Another recent study in mice confirms our results, indicating that prenatal exposure to VPA causes a significant decrease in the number of microglia in the primary motor cortex in the early stages of brain development: this early decrease in microglia led the authors to hypothesise that dysregulated cortical synaptogenesis may play a role in the aetiopathogenesis of ASD [102].

In this study, VPA dose–response effects on numerous biological features were observed in zebrafish, and two different phenotypes of interest for ASD research were identified and extensively investigated. A mild phenotype was characterised by a slight delay in body growth, but neither morphological and mitochondrial defects nor alterations in neurodevelopmental or inflammation-related gene expression were identified. In this mild phenotype, only a general impairment of locomotor and visual activity was highlighted, along with a reduction in resident microglia. The second identified phenotype (obtained by doubling the VPA dosage) displayed more severe features, namely a greater delay in somatic, nervous, and visual system development and significant impairment of both mitochondrial energy metabolism and ROS accumulation. Furthermore, gene expression investigations and microglial assessments revealed a marked pro-inflammatory state in moderate-phenotype individuals. These debilitating biological alterations led to reduced locomotor and visual activity in response to sensory stimuli. It should also be noted that VPA, at all the doses administered in the present study, caused an increased heart rate in this animal model.

This study is subject to some limitations that could be addressed in future research. For example, given the impaired locomotor behavioural response associated with the higher dose of VPA administered, it might be useful to perform molecular investigations focusing on neurotransmitter analysis. Furthermore, it could be interesting to analyse motor neuron function, with the aim of shedding light on mechanisms of VPA-induced functional impairment.

## 5. Conclusions

In conclusion, our study provides comprehensive insights into the neurodevelopmental and behavioural effects of VPA exposure in zebrafish larvae, highlighting its role in modelling ASD-like phenotypes. By assessing a wide range of parameters, including locomotor behaviour, neuroinflammatory markers, gene expression profiles, mitochondrial function, and cardiac and visual responses, we were able to highlight the multifaceted impact of VPA on embryonic development. Our findings support the notion that VPA-induced alterations in neurodevelopmental processes, including microglial activation, oxidative stress, and dysregulated gene expression, may have implications for elucidating the mechanisms involved in the pathogenesis of ASD. Moreover, the observed cardiac and visual impairments highlight the effects of VPA exposure on various organ systems. The ASD phenotypes identified and characterised here could become useful tools for ASD research and drug/nutraceutical screening studies. These results also reinforce the usefulness of zebrafish models in studying the complex interplay between genetic and environmental factors in neurodevelopmental disorders. Future studies are essential to identify novel ASD biomarkers and to develop both new drug targets and tailored therapeutic interventions for ASD. 

## Data Availability

The original contributions presented in the study are included in the article/Supplementary Materials; further inquiries can be directed to the corresponding authors.

## Supplementary Materials

The following supporting information can be downloaded at: https://www.mdpi.com/2073-4409/13/16/1349/s1, Figure S1: Anatomical brain regions of interest (ROIs) selected to quantify microglia: the red ROI is in the telencephalon, the yellow ROIs are in the optic tectum, and the blue ROIs are in the hindbrain; Figure S2: OKR analysis. (A) Larvae positioning; (B) eye orientation for detection of saccades; (C) digital vertical stripes projected in the Visiobox drum; Table S1: qPCR primers used for gene expression analysis.
