# Supplementary material for: Delving into the Complexity of Valproate-Induced Autism Spectrum Disorder: The Use of Zebrafish Models
Source: Cells. 2024 Aug 14;13(16):1349. doi: 10.3390/cells13161349 (PMC11487397; doi:10.3390/cells13161349)
Supplement: Supplementary file 1 [file cells-13-01349-s001.zip › Supplementary Figures.pdf]

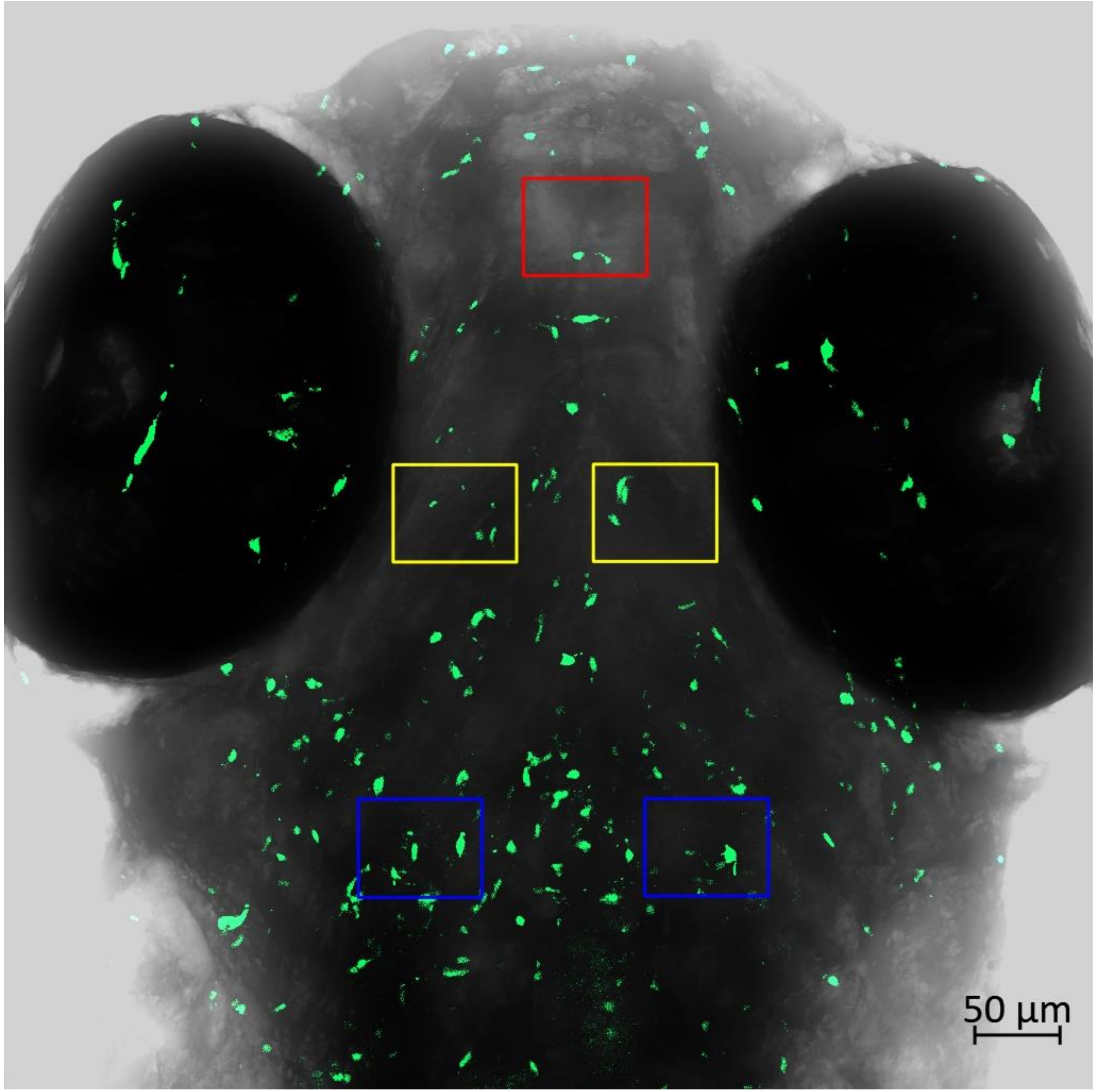

Supplementary Figure S1. – Anatomical brain regions of interest (ROIs) selected to quantify microglia: the red ROI is in the telencephalon, the yellow ROIs are in the optic tectum, and the blue ROIs are in hindbrain.

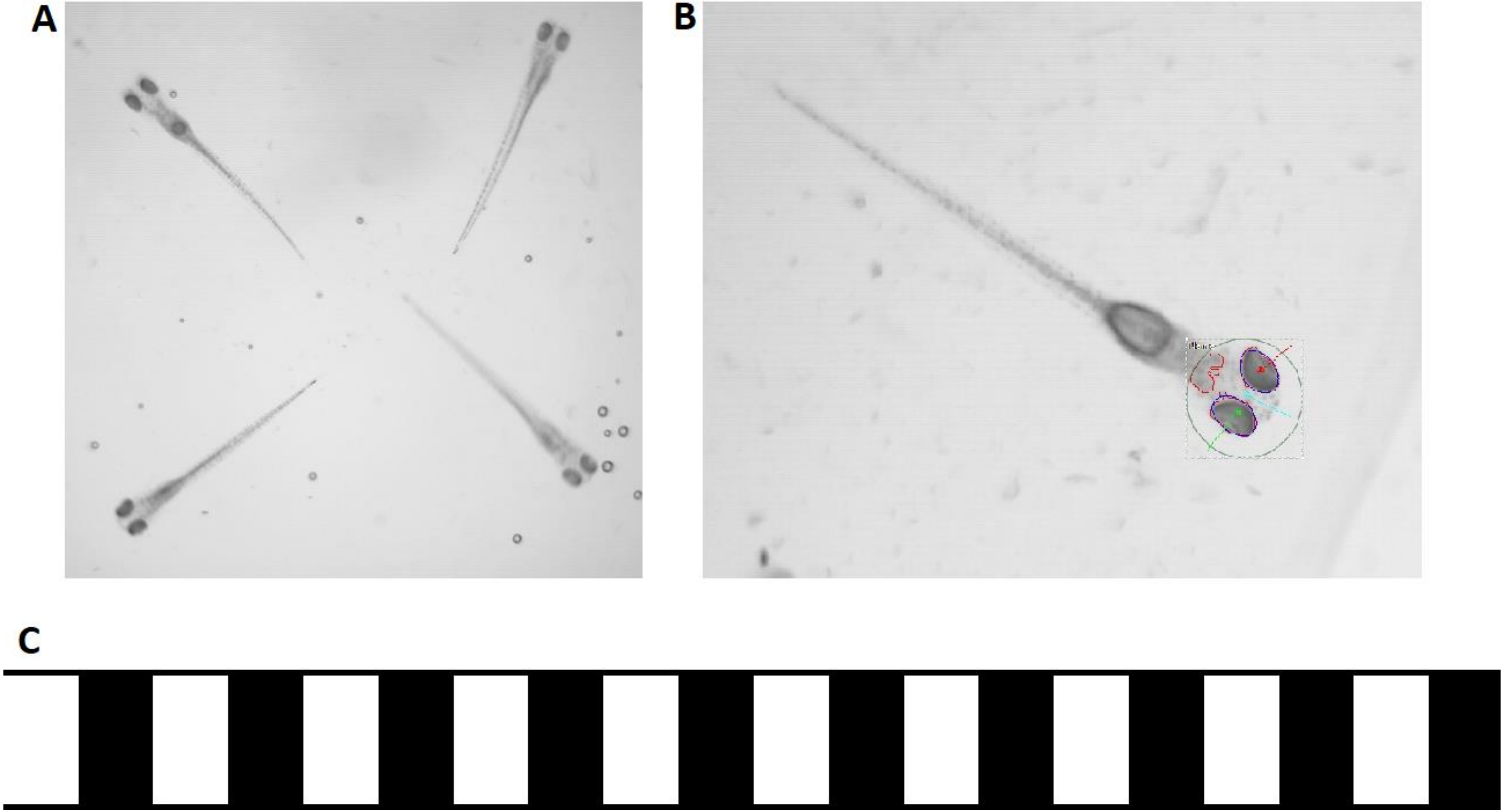

Supplementary Figure S2. – OKR analysis. **(A)** Larvae positioning;
(B) Eye orientation for detection of saccades; **(C)** Digital vertical stripes projected in the Visiobox drum
