# Supplementary material for: Delving into the Complexity of Valproate-Induced Autism Spectrum Disorder: The Use of Zebrafish Models
Source: Cells. 2024 Aug 14;13(16):1349. doi: 10.3390/cells13161349 (PMC11487397; doi:10.3390/cells13161349)
Supplement: Supplementary file 1 [file cells-13-01349-s001.zip › Supplementary Table S1.pdf]

Table S1: qPCR primers used for gene expression analysis

**Genes**	**Sequence 5'-3'**
β-actin - F	GCAGAAGGAGATCACATCCCTGGC
β-actin - R	CATTGCCGTCACCTTCACCGTTC
IL-1β - F	GGCAACTGTTCCTGAACTCAACTG
IL-1β - R	CCATTGAGGTGGAGAGCTTTCAGC
IL-6 - F	CCACTTCACAAGTCGGAGGCTT
IL-6 - R	CCAGCTTATCTGTTAGGAGA
IL-4 - F	TCTCTGCCAAGCAGGAATG
IL-4 - R	CAGTTTCCAGTCCCGGTATATG
HDAC-4 - F	GAGACGGGGAATGTGAAAGAC
HDAC-4 - R	TGTTTCTGCTTGAGGGCCAG
NRXN-1 - F	GAGCAGTAGCGATGAGATTAC
NRXN-1 - R	ACTACCGCCGACATAGAA
NGLN-3 - F	CTGCTGACTCTTTCCCATTAT
NGLN-3 - R	CCTGCTCCACTAGTTCTTTG
NGF - F	TTTGCCACCTGGAATGCAAC
NGF - R	GCCGAAGTACGAGATTCCCAT
IL-10 - F	CACAACCCCAATCGACTCCA
IL-10 - R	GGCAAGAAAAGTACCTCTTGCAT
C-FOS - F	CACCGATACACTCGAAGCTGAA
C-FOS - R	CAGGTTGGCGATGTCGTTCT
